# Reshaping the landscape of HLA desensitization through immune engineering

**DOI:** 10.3389/ti.2026.17609

**Published:** 2026-09-14

**Authors:** Delphine Kervella, Oriol Bestard

**Affiliations:** 1 Kidney Transplant Unit, Nephrology Department, Vall d’Hebron Hospital Universitari, Vall d’Hebron Institut de Recerca (VHIR), Vall d’Hebron Barcelona Hospital Campus, Universitat Autònoma de Barcelona, Barcelona, Spain; 2 Laboratory of Nephrology and Transplantation, Vall d'Hebron Institut de Recerca (VHIR), Vall d’Hebron Barcelona Hospital Campus, Universitat Autònoma de Barcelona, Barcelona, Spain; 3 Redes de Investigación Cooperativa Orientadas a Resultados en Salud (RICORS), Barcelona, Spain

**Keywords:** access to transplantation, CAR T cells, desensitization, HLA allosensitization, kidney transplantation

Kidney transplantation in highly HLA-sensitized candidates remains one of the greatest unmet challenges in transplantation. Since transplantation with donor specific antibodies (DSA) is associated with an increased risk of rejection and graft loss [1], strategies to enable HLA compatible transplantation should be encouraged, such as kidney exchange programs and different prioritization programs [2]. However, despite major advances in histocompatibility testing and allocation policies prioritizing sensitized recipients, patients with broad sensitization (cPRA >99%) continue to experience prolonged waiting times, low access to compatible donors, and increased mortality while on the waiting list [3–5]. Consequently, HLA desensitization has emerged as the only viable option for transplantation in many of these patients.

Over the past decades, numerous desensitization strategies have sought to overcome HLA sensitization and expand access to transplantation. However, most have relied on off-label immunosuppressive therapies targeting isolated components of the humoral immune response, circulating antibodies, plasma cells, or memory B cells, rather than the integrated biology of alloimmune memory. Consequently, clinical efficacy has been inconsistent and durable desensitization has remained largely elusive [6, 7].

The introduction of imlifidase represented the first major breakthrough by enabling rapid elimination of circulating donor-specific antibodies (DSA) and successful HLA-incompatible transplantation in selected highly sensitized recipients [8–10]. Although encouraging short- and intermediate-term outcomes have been reported, its long-term impact remains uncertain because rapid antibody rebound, driven by persistent memory B cells and long-lived plasma cells, frequently occurs within days to weeks after transplantation [11, 12]. Thus, while imlifidase established proof-of-concept that deeply entrenched humoral alloimmunity can be therapeutically overcome, it also highlighted the need for strategies capable of durably targeting the cellular reservoirs that sustain HLA antibody production.

Three reports published in the New England Journal of Medicine [13–15] provide compelling proof-of-concept that immune-engineering approaches may substantially expand the therapeutic armamentarium for HLA desensitization. The remarkable efficacy of chimeric antigen receptor (CAR)-T cells and T-cell engager (TCE) bispecific antibodies targeting B-cell and plasma-cell lineages in hematologic malignancies, together with their emerging success in severe refractory autoimmune diseases [16, 17], has now prompted their translation into transplantation. Four highly sensitized kidney transplant candidates (cPRA >99.8%) underwent successful desensitization followed by HLA-compatible kidney transplantation after receiving different immune-engineering platforms targeting complementary cellular compartments through CD19, B-cell maturation antigen (BCMA), or both. Rather than simply removing circulating donor-specific antibodies (DSAs), these pioneering approaches aim to eradicate the cellular reservoirs responsible for sustaining humoral alloimmune memory. Although based on only a handful of patients, these reports provide an important biological proof-of-concept and establish a strong rationale for future clinical investigation.

Direct comparison among these studies is challenging because they employed distinct therapeutic platforms (one CD19 CAR-T, two dual CD19/BCMA CAR-T, and one CD3×BCMA TCE), different cell doses, and variable lymphodepletion regimens. Nevertheless, all strategies produced profound reductions in circulating HLA antibodies, enabling timely HLA-compatible kidney transplantation. Perhaps even more strikingly, none of the treated patients experienced DSA rebound following transplantation despite re-exposure to donor HLA antigens, suggesting that these approaches may interfere with the immunological mechanisms underlying anamnestic humoral responses. This observation is particularly intriguing for strategies primarily targeting only one of the two principal compartments sustaining alloantibody production (memory B cells or long-lived plasma cells) as compensatory responses arising from the untargeted compartment have previously been described with other, predominantly monoclonal antibody-based, desensitization approaches [18, 19]. Indeed, these observations are consistent with a recent desensitization experience using a BCMA-directed TCE combined with obinutuzumab, a next-generation anti-CD20 monoclonal antibody, which similarly induced marked reductions in HLA antibodies, enabled successful transplantation, and maintained durable biological effects after transplantation [20].

Importantly, despite the intensity of these immune interventions, all reports describe an acceptable short-term safety profile. Treatment-related toxicities were limited, with only one patient receiving high-dose CD19 CAR-T therapy developing mild grade I cytokine release syndrome (CRS), no cases of immune effector cell-associated neurotoxicity syndrome (ICANS), and no major opportunistic infections either during dialysis or after transplantation under conventional induction and maintenance immunosuppression. However, all patients developed profound hypogammaglobulinemia due to depletion of polyclonal immunoglobulins, necessitating prolonged intravenous immunoglobulin replacement in most cases.

Beyond their clinical findings, these reports provide valuable mechanistic insights into the biology of HLA antibody persistence. Serial antibody measurements illustrate the extraordinary burden of circulating HLA antibodies in highly sensitized patients and suggest that several physiological antibody turnover cycles are required before complete clearance becomes evident. This was particularly evident for anti-HLA-DQ antibodies, consistent with their typically higher circulating antibody burden in highly sensitized recipients. Furthermore, in patients who underwent a single immunoadsorption session before transplantation, a marked reduction in HLA antibody levels was achieved without subsequent rebound, further supporting the notion that antibody production had been effectively suppressed rather than merely transiently reduced.

Comprehensive immune phenotyping further supports the proposed mechanisms of action. Peripheral blood B cells were completely depleted in all patients throughout follow-up, except in the two recipients of dual CD19/BCMA CAR-T cells, in whom B-cell reconstitution occurred after approximately 53 days. Bone marrow analyses demonstrated effective depletion of CD19-positive cellular populations across all strategies, whereas profound elimination of bone marrow-resident plasma cells was observed only in patients receiving BCMA-targeted therapies. Likewise, lymph node analyses showed marked depletion of B-cell populations following both CD19-only and dual CD19/BCMA CAR-T therapies, although comparable tissue analyses were unfortunately unavailable in the patient treated with the CD3×BCMA bispecific antibody. In this line, antibodies directed against previously encountered HLA specificities appeared more resistant to depletion following CD19 CAR-T therapy. In contrast, no preferential persistence of antibodies against repeated donor HLA mismatches was observed in the patient receiving BCMA-targeted therapy despite antigen rechallenge at kidney transplantation. Although based on a single case, these observations suggest that the durability of established alloantibody responses may depend on the underlying mechanisms sustaining humoral immune memory, including the relative contribution of memory B cells, long-lived plasma cells, and persistent antigenic stimulation.

Collectively, these pioneering studies provide compelling proof-of-concept that immune-engineering approaches may reshape the landscape of HLA desensitization. However, many fundamental questions remain. Which therapeutic platform, or combination of cellular targets, provides the optimal balance between efficacy and safety? The slower decline of anti-HLA-DQ antibodies, likely reflecting their particularly high antibody burden, together with the persistence of antibodies directed against previously encountered HLA antigens after some treatment approaches, illustrates that distinct components of humoral alloimmunity may differ in their susceptibility to these new immune engineering therapies (Table 1). Whether depletion of memory B cells alone is sufficient, or sustained plasma-cell depletion is also required to prevent anamnestic alloantibody responses, remains unknown. Addressing these questions will require adequately powered prospective clinical trials comparing these different strategies, which should invariably be accompanied by comprehensive mechanistic studies to define which cellular compartments must be effectively targeted for durable control of humoral alloimmunity. Equally important, the long-term consequences of prolonged hypogammaglobulinemia, the optimal post-transplant immunosuppressive strategy, and the risk of opportunistic viral infections remain to be established before these transformative approaches can be broadly implemented. Ultimately, lessons from these emerging immune-engineering platforms may pave the way toward HLA-specific desensitization, selectively eliminating pathogenic alloimmune memory while preserving protective immunity, likely complemented by non–antigen-specific plasma cell–directed therapies [21–23].

**TABLE 1:** Main goals and biological features of different desensitization strategies.

Feature	Conventional desensitization (PLEX/IVIG ± anti-CD20)	Imlifidase	CD19 CAR-T	CD19/BCMA CAR-T	BCMA T-cell engager (TCE)
Primary humoral immune compartment targeted	Circulating antibodies + partial B-cell depletion	Circulating IgG antibodies	CD19^+^ B-cell lineage (including naïve, memory B cells and some plasma cells; largely sparing CD19^−^ long-lived plasma cells)	CD19^+^ B-cell lineage + BCMA^+^ plasma cells (including long-lived plasma cells)	BCMA^+^ plasma cells (sparing BCMA^−^ memory B cells)
HLA antibody kinetics	Partial and variable reduction	Immediate, complete but transient elimination	Progressive decline	Progressive, potentially more sustained decline	Progressive decline
Access to HLA-compatible transplantation	Limited	✓	✓	✓	✓
Observed DSA rebound	Frequent	Frequent	None observed*	None observed*	None observed*
Principal biological limitation	Incomplete control of humoral alloimmunity	Persistence of humoral immune memory	Persistence of CD19^−^ long-lived plasma cells	Prolonged hypogammaglobulinemia and immune deficiency	Persistence of memory B cells
Major unanswered question	—	How can antibody rebound be prevented?	Is depletion of CD19^+^ B-cell compartments sufficient for durable desensitization?	Does dual targeting provide superior durability that justifies deeper immune depletion?	Is plasma-cell depletion alone sufficient without eliminating memory B cells?
Conceptual advance	Suppress humoral alloimmunity	Remove circulating antibodies	Reprogram humoral immune memory	Reprogram humoral immune memory	Reprogram humoral immune memory

* No DSA, rebound has been observed during the available follow-up. However, long-term durability remains unknown and will likely depend on both the completeness of depletion of the relevant humoral immune compartments and the nature of subsequent HLA, antigen re-exposure, including repeated HLA, antigen encounters, shared epitopes, and persistent antigen expression from retained failed allografts.

## Data Availability

The original contributions presented in the study are included in the article/supplementary material, further inquiries can be directed to the corresponding author.
